# Metabolic engineering of *Bacillus subtilis* for chiral pure meso-2,3-butanediol production

**DOI:** 10.1186/s13068-016-0502-5

**Published:** 2016-04-19

**Authors:** Jing Fu, Guangxin Huo, Lili Feng, Yufeng Mao, Zhiwen Wang, Hongwu Ma, Tao Chen, Xueming Zhao

**Affiliations:** Key Laboratory of Systems Bioengineering (Ministry of Education); SynBio Research Platform, Collaborative Innovation Center of Chemical Science and Engineering (Tianjin), School of Chemical Engineering and Technology, Tianjin University, Tianjin, 300072 People’s Republic of China; Key Laboratory of Fermentation Engineering (Ministry of Education), Hubei Provincial Cooperative Innovation Center of Industrial Fermentation, Hubei University of Technology, Wuhan, 430068 China; Key Laboratory of Systems Microbial Biotechnology, Tianjin Institute of Industrial Biotechnology, Chinese Academy of Sciences, Tianjin, 300308 People’s Republic of China

**Keywords:** Metabolic engineering, Meso-2,3-butanediol, d-(−)-2,3-butanediol, *Bacillus subtilis*, Cofactor engineering

## Abstract

**Background:**

2,3-Butanediol (2,3-BD) with low toxicity to microbes, could be a promising alternative for biofuel production. However, most of the 2,3-BD producers are opportunistic pathogens that are not suitable for industrial-scale fermentation. In our previous study, wild-type *Bacillus subtilis* 168, as a class I microorganism, was first found to generate only d-(−)-2,3-BD (purity >99 %) under low oxygen conditions.

**Results:**

In this work, *B. subtilis* was engineered to produce chiral pure meso-2,3-BD. First, d-(−)-2,3-BD production was abolished by deleting d-(−)-2,3-BD dehydrogenase coding gene *bdhA*, and *acoA* gene was knocked out to prevent the degradation of acetoin (AC), the immediate precursor of 2,3-BD. Next, both *pta* and *ldh* gene were deleted to decrease the accumulation of the byproducts, acetate and l-lactate. We further introduced the meso-2,3-BD dehydrogenase coding gene *budC* from *Klebsiellapneumoniae* CICC10011, as well as overexpressed *alsSD* in the tetra-mutant (Δ*acoA*Δ*bdhA*Δ*pta*Δ*ldh*) to achieve the efficient production of chiral meso-2,3-BD. Finally, the pool of NADH availability was further increased to facilitate the conversion of meso-2,3-BD from AC by overexpressing *udhA* gene (coding a soluble transhydrogenase) and low dissolved oxygen control during the cultivation. Under microaerobic oxygen conditions, the best strain BSF9 produced 103.7 g/L meso-2,3-BD with a yield of 0.487 g/g glucose in the 5-L batch fermenter, and the titer of the main byproduct AC was no more than 1.1 g/L.

**Conclusion:**

This work offered a novel strategy for the production of chiral pure meso-2,3-BD in *B. subtilis*. To our knowledge, this is the first report indicating that metabolic engineered *B. subtilis* could produce chiral meso-2,3-BD with high purity under limited oxygen conditions. These results further demonstrated that *B. subtilis* as a class I microorganism is a competitive industrial-level meso-2,3-BD producer.

**Electronic supplementary material:**

The online version of this article (doi:10.1186/s13068-016-0502-5) contains supplementary material, which is available to authorized users.

## Background

2,3-Butanediol (2,3-BD) is an important platform compound, and its derivative products are also promising valuable chemicals due to their extensive industrial applications [1, 2]. 2,3-BD can exist in three isomeric forms: d-(−)-, L-(+)- and meso-, and the chiral stereoisomers of 2,3-BD can be used as the precursor for the synthesis of various chiral compounds or intermediates, which can increase the value of the product [3]. Considering the high cost in chemical synthesis and separation steps [3], chiral pure 2,3-BD stereoisomers produced by engineered strains have gained much more interest in recent years.

Microorganisms can produce a mixture of 2,3-BD stereoisomers [4]. The ratio varies dramatically depending on the species, and especially the fermentation conditions such as oxygen-diluted rate, temperature, pH, and substrate type [5]. In the recent years, research interest in microbial 2,3-BD production has increased significantly. *Klebsiella pneumoniae* [6, 7], *K. oxytoca* [8, 9], and *Enterobacter aerogenes* [10–12] are considered to be absolutely unbeatable in terms of 2,3-BD production efficiency, which can produce a mixture of meso- and L-(+)-stereoisomers of 2,3-BD with a ratio of approximately 9:1 [2]. *Serratia marcescens* [13, 14] is also considered to be a promising microorganism that can produce meso- and d-(−)- stereoisomers of 2,3-BD (primarily meso-2,3-BD). Recently, *Bacillus amyloliquefaciens* was engineered to produce 132.9 g/L 2,3-BD with a productivity of 2.95 g/(L h) form glucose [15], and 102.3 g/L from biodiesel-derived glycerol [16]. However, all the above mentioned strains are pathogenic microorganisms [1], which might be hazardous to human use and increase the cost of industrial-scale production. As for industrially friendly hosts, *Escherichia coli* was optimized to produce 73.8 g/L meso-2,3-BD with a yield of 0.41 g/g glucose [17], and 54 g/L meso-2,3-BD from glucose and xylose mixture with a productivity of 0.45 g/(L h) using biomass-inducible chromosome-based expression system [18]. In a recent study, *E. coli* was engineered to produce d-(−)-2,3-BD with a titer of 115 g/L with high purity [19]. In addition, *Saccharomyces cerevisiae* was engineered to produce 72.9 g/L from glucose with a yield of 0.41 g/g glucose [20], and more than 100 g/L d-(−)-2,3-BD from a mixture of glucose and galactose in up to 300 h [21].

*Bacillus subtilis*, which was granted GRAS (generally regarded as safe) status by the US Food and Drug Administration [22], has been applied as a model system for researchers on the aspects of biochemistry [23], genetics, and physiology of gram-positive bacteria. It has long been widely used for the environmental, medical, and industrial applications [24]. In our recent study, wild-type *B. subtilis* 168 was found to generate only d-(−)-2,3-BD (purity >99 %) under low oxygen conditions [25].

As the direct precursor of 2,3-BD, acetoin (AC) formation from pyruvate was catalyzed in a two-step reaction by acetolactate synthase and acetolactate decarboxylase in *B. subtilis*, coded by *alsS* and *alsD*, respectively [26]. It was indicated that although the LysR-type transcriptional regulator AlsR protein (coded by *alsR*) positively regulated the *alsSD* operon [27, 28] in *B. subtilis*, it indirectly regulated the expression of *bdhA* (encoding 2,3-BD dehydrogenase/AC reductase) [29]. The biodegradation of AC was catabolized by the AC dehydrogenase enzyme system, and its core subunit encoded by *acoA* affects the dehydrogenase complex activity significantly [30]. AC was reduced to 2,3-BD by 2,3-BD dehydrogenase (BDH), also called AC reductase (AR), with oxidation of NADH to NAD^+^. The regulation facilitating increased NADH availability and NADH/NAD^+^ ratio played a key role for the enhanced 2,3-BD/AC production [25, 31]. In our previous study, the production of AC was enhanced by insertional inactivation of the *acoA* and *pta* genes, and overexpression of *alsS* and *alsD* [32, 33]. In this work, the lactate dehydrogenase coding gene *ldh* was deleted to reduce the byproduct lactate and increase the NADH availability for enhanced meso-2,3-BD production.

Dissolved oxygen (DO) level had been shown to have a profound effect on the product distribution, with AC being excreted with DO greater than 100 parts per billion (ppb) and 2,3-BD, less than 100 ppb [34]. The product concentration ratio (2,3-BD to AC) changed rapidly in the 80–90 ppb range [35]. The expressions of *alsS* and *alsD* were increased under anaerobic conditions compared with aerobic condition, indicating that 2,3-BD might be accumulated to a higher level when DO decreased to a certain degree [29, 36]. In a recent study, the initial concentration of corn steep liquor had remarkable effects on both 2,3-BD production and the ratio of 2,3-BD to AC [37].

**Fig. 1:**
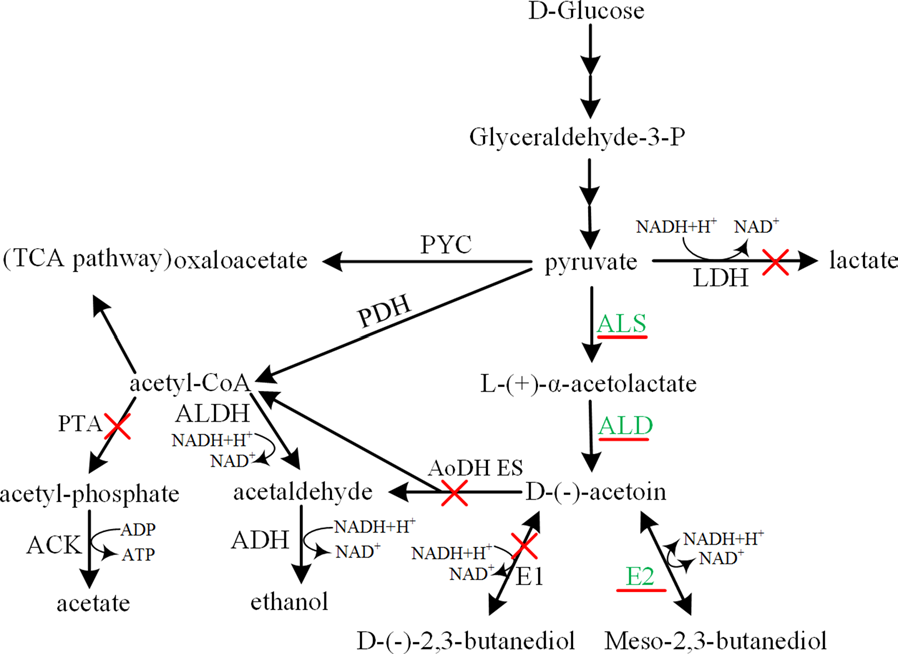
The meso-2,3-BD biosynthetic pathway in *B. subtilis*. Enzymes overexpressed or introduced are underlined and depicted in *green*, and those interrupted are shown in *red* with a *cross*. Expanded names of enzymes and their coding genes: ALS acetolactate synthase, *alsS*; ALD acetolactate decarboxylase, *alsD*; ACK acetate kinase, ack; ALDH acetaldehyde dehydrogenase, *adh*; ADH alcohol dehydrogenase, *ack*; LDH lactate dehydrogenase, *ldh*; PDH pyruvate dehydrogenase, *pdhABCD*; PTA phosphotransacetylase, *pta*; PYC pyruvate carboxylase; E1, d-(−)-butanediol dehydrogenase (EC 1.1.1.4), *bdhA*; E2, L-(+)-butanediol dehydrogenase (EC 1.1.1.76), *budC*

In this work, a series of mutant strains were constructed to improve the yield and production of meso-2,3-BD step by step, including disruption of all the target genes (*acoA*, *bdhA*, *pta*, and *ldh*), introduction of meso-2,3-butadediol coding gene *budC* from *K. pneumoniae* CICC10011, and overexpressions of *alsS* and *alsD* in 2,3-BD synthetic pathway (Fig. 1) by means of a modified marker-less genetic manipulation system [38, 39]. In addition, deletion of *ldh* and overexpression of *udhA* were employed to improve the availability of NADH for AC reductase (AR). Batch cultures under microaerobic conditions were employed to investigate the performances of these genetically modified *B. subtilis* mutants. Finally, strain BSF9 produced 103.7 g/L meso-2,3-BD (purity >99 %) with a yield of 0.487 g/g glucose in fed-batch experiment.

## Results and discussion

### Increasing metabolic flux in 2,3-BD pathway by reducing AC consumption and eliminating byproducts

With the purpose of increasing AC, the supply of the direct substrate of 2,3-BD, *acoA* was disrupted in BSF1 (*B. subtilis* 168Δ*upp*), yielding BSF2. The growth and products of the mutants were characterized and are summarized in Table 1. When glucose was depleted, the deletion led to a slight increase of total production of 2,3-BD and AC in BSF2 (2.84 g/L BD + 1.03 g/L AC) compared to that of BSF1 (2.70 g/L BD + 0.82 g/L AC). Unlike previous report [30] in which AC was mainly consumed by AC dehydrogenase enzyme pathway after glucose was exhausted, this result demonstrated that partial AC had already been consumed before glucose was exhausted. After glucose was exhausted, the deletion of *acoA* also reduced the conversion of AC to acetate as previously reported [26]. BSF1 consumed about 1.2 g/L AC, as well as produced about 0.3 g/L acetate from 60 h to 96 h (data not shown). By analysis of extracellular metabolites by HPLC, we found that acetate was the main product of the oxidative dissimilation of AC in *B. subtilis*, which is consistent with Lopez et al.’s study on the direct oxidative cleavage of AC [40]. In the case of the *acoA*-deleted strain BSF2 during this period, however, the concentration of AC was reduced by only 0.25 g/L (2.8 mM) (data not shown). These results implied that the deletion of *acoA* could lead to the increasing accumulation of AC, and further increase in its direct production of 2,3-BD.

**Table 1:** Metabolic characterizations of *B. subtilis* strains cultivated in M9 medium supplemented with 10 g/L glucose under the microaerobic condition

Strain	CDW^a,c^ (g/L)	2,3-BD^b,c^ (g/L)	AC^b,c^ (g/L)	Acetate^b,c^ (g/L)	Lactate^b,c^ (g/L)	Succinate^b^ (g/L)	Glucose uptake rate^c^ [g/(L h)]	Time for glucose depletion^c^ (<0.2 g/L) (h)	2,3-BD productivity^c^ (g/(L h))	2,3-BD yield^c^ (mol/mol)	AC + 2,3-BD (at 60 h) (g/L)	AC + 2,3-BD (at 96 h) (g/L)
BSF1^c^	0.960 ± 0.012	2.70(D) ± 0.05	0.82 ± 0.06	0.24 ± 0.03	0.40 ± 0.04	0.08 ± 0.00	0.270	37	0.073	0.54	4.10 + 0.27	2.90 + 0
BSF2	0.920 ± 0.005	2.84(D) ± 0.11	1.03 ± 0.07	0.20 ± 0.02	0.36 ± 0.01	0.09 ± 0.01	0.260	38.5	0.074	0.57	4.20 + 0.19	3.95 + 0
BSF3^c^	0.898 ± 0.015	ND^d^	3.83 ± 0.02	0.17 ± 0.01	0.44 ± 0.03	0.07 ± 0.00	0.233	43	0	0	4.21 + 0	4.00 + 0
BSF4	0.828 ± 0.011	ND	3.70 ± 0.05	0.14 ± 0.01	0.34 ± 0.05	0.11 ± 0.01	0.222	45	0	0	3.87 + 0	3.64 + 0
BSF5	0.814 ± 0.009	ND	3.71 ± 0.10	0.71 ± 0.01	ND	0.29 ± 0.01	0.208	48	0	0	3.69 + 0	3.50 + 0
BSF6	0.806 ± 0.004	ND	3.88 ± 0.02	0.61 ± 0.01	ND	0.20 ± 0.03	0.200	50	0	0	3.71 + 0	3.52 + 0
BSF7	0.813 ± 0.007	2.85(M) ± 0.12	0.91 ± 0.11	0.73 ± 0.03	ND	0.28 ± 0.02	0.244	41	0.070	0.57	2.14 + 1.83	2.04 + 0.97
BSF8	0.838 ± 0.010	2.90(M) ± 0.05	1.10 ± 0.02	0.65 ± 0.01	ND	0.25 ± 0.02	0.244	41	0.071	0.58	1.93 + 1.58	1.90 + 0.88
BSF9	0.846 ± 0.008	3.56(M) ± 0.08	0.68 ± 0.05	0.56 ± 0.04	ND	0.19 ± 0.01	0.256	39	0.091	0.71	1.70 + 2.43	1.72 + 1.93
BSF17	0.970 ± 0.008	2.93(M) ± 0.09	1.04 ± 0.08	0.18 ± 0.02	0.66 ± 0.03	0.05 ± 0.00	0.270	37	0.079	0.59	2.42 + 1.56	2.51 + 0.81
BSF18^c^	0.945 ± 0.009	3.01(D) ± 0.06	0.92 ± 0.09	0.29 ± 0.01	0.69 ± 0.03	0.06 ± 0.00	0.260	38	0.079	0.60	3.92 + 0.11	3.85 + 0
BSF25	0.969 ± 0.012	3.28(M) ± 0.04	0.85 ± 0.07	0.14 ± 0.01	0.25 ± 0.02	0.06 ± 0.00	0.278	36	0.091	0.66	2.96 + 1.31	2.53 + 0.94

Bacteria were cultivated with 100 mL M9 in a 250-mL flask at an agitation speed of 100 rpm and 37 °C; Data are average values and standard deviations of triplicate experiments. CDW cell dry weight, 2,3-BD 2,3-butanediol, D d-(−)-2,3-BD, M meso-2,3-BD; ND not detected or lower than 0.01 g/L

^a^The CDW was calculated using the relationship “CDW = OD_600_*0.325”

^b^These metabolites were measured when the residual glucose almost reached zero (<0.2 g/L)

^c^These results for BSF1, BSF3, and BSF18 were published in a previous paper (Fu et al. [25]), and are listed here for relative discussion in this paper

**Fig. 2:**
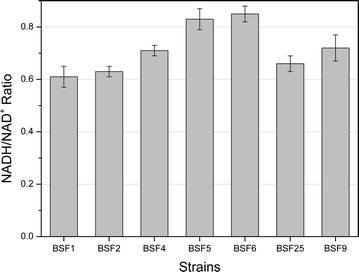
Changes in the levels of intracellular NADH/NAD^+^ ratios in different strains. Bacteria were cultivated using a mixture of 100 mL M9 and 10 g/L glucose in a 250-mL flask kept agitated at a speed of 100 rpm and 37 °C. The intracellular NADH and NAD^+^ were extracted after 30 h. Data show average values and standard deviations of triplicate experiments

As shown in Table 1, a small amount of acetate had been detected in the media of strains BSF2 when glucose was depleted, while lactate and succinate accumulated with maximum concentrations of 0.36 and 0.09 g/L, respectively. To eliminate the accumulation of byproducts, acetate and lactate, *pta* (coding phosphate acetyltransferase) and *ldh* (coding l-lactate dehydrogenase) were deleted, respectively. Inactivation of *pta* had almost no effect on acetate reduction of BSF4 (0.14 ± 0.01 g/L) compared with BSF3 (*bdhA* deleted in BSF2 for abolishing of d-(−)-2,3-BD) (0.17 ± 0.01 g/L), indicating there might be other major acetate or acetyl-phosphate synthetic pathways in *B. subtilis* [41]. However, the biomass decreased from 0.898 g/L DCW for BSF3 to 0.828 g/L DCW for BSF4, while the glucose consumption rate slightly decreased from 0.233 g/(L h) of BSF3 to 0.222 g/(L h) of BSF4. The disruption of *ldh* abolished lactate accumulation of BSF5; however, it drastically increased the formations of acetate and succinate to 0.71 and 0.29 g/L, respectively. The increasing concentrations of acetate and succinate might presumably have resulted from an unknown regulatory mechanism that affected the other acetate synthesis pathway of *ldh*-deleted mutant BSF5. In our previous study, we observed that *bdhA* deletion resulted in a lower biomass (0.960 g/L for BSF1 versus 0.898 g/L for BSF3) and a slower glucose consumption rate [0.270 g/(L h) for BSF1 versus 0.233 g/(L h) for BSF3]. Here, we found that the knockout of *ldh* in *bdhA*-deleted mutant BSF4 further decreased the biomass formation (from 0.828 to 0.814 g/L) and glucose consumption rate [from 0.222 to 0.208 g/(L h)]. It was also reported that deletion of *ldh* in *B. subtilis* resulted in drastic growth reduction under anaerobic conditions [42]. All these results might be ascribed to the fact that blocking the biosynthesis pathways of reducing metabolites caused the imbalance of reducing power to some extent, which was demonstrated by the intracellular NADH/NAD^+^ analysis (Fig. 2). The NADH/NAD^+^ level increased from 0.61 for BSF1 to 0.71 for BSF4, and further increased to 0.83 for BSF5. For all the strains containing *ldh*, lactate was accumulated to its highest concentration when glucose was depleted and was quickly reused when glucose was exhausted, indicating that lactate might be a direct carbon source for *B. subtilis* after the glucose was depleted in minimal medium. Although deletion of *ldh* did not increase metabolic flux in 2,3-BD pathway, it could increase the NADH/NAD^+^ ratio for further conversion of AC to 2,3-BD, as the NADH availability was the key factor for 2,3-BD production [25].

### Enhancing AC synthetic pathway

For the purpose of increasing metabolic flux in AC synthetic pathway, an additional copy of *alsSD* mediated by a strong promoter *P*_*43*_ [43] was integrated into the chromosome of BSF5 at *bdhA* locus, yielding BSF6. The additional copies of *alsS* and *alsD* in BSF6 were constitutively expressed without the regulation of *alsR* [29, 36]. The yield of AC of BSF6 was almost unchanged; however, concentrations of acetate and succinate of BSF6 (0.61 g/L and 0.20 g/L, respectively) decreased slightly compared to those of BSF5 (0.71 and 0.29 g/L, respectively). Indeed, the overexpressions of *alsS* and *alsD* did not contribute much to AC production (3.71 g/L of BSF5 versus 3.88 g/L of BSF6), probably because the activities of the two enzymes in *B. subtilis* were high enough, and the *alsS*–*alsD* pathway played the dominating role in the metabolism. As a result, the most competitive strain BSF6 with the highest AC production (Table 1), high NADH/NAD^+^ ratio, and abolishing of d-(−)-2,3-BD accumulation was used as the parent strain for further construction of meso-2,3-BD producer.

### Introducing meso-2,3-BD dehydrogenase for chiral meso-2,3-BD production

To produce chiral meso-2,3-BD, *budC* from *Klebsiella pneumoniae* CICC10011 was introduced in BSF5 and BSF6, yielding BSF7 and BSF8, respectively. BSF7 produced 2.85 g/L meso-2,3-BD and 0.91 g/L AC, while *alsSD*-overexpressing strain BSF8 produced slightly more meso-BD and AC (2.90 and 1.10 g/L, respectively). As the major byproducts, the concentrations (Table 1) of acetate and succinate of BSF8 were slightly lower than those of BSF7, which was similar to the results of BSF6 with *alsSD* overexpressed. The glucose consumption rate was partially restored by overexpression of *budC*, indicating that excess NADH, which might impact the glucose consumption rate of BSF6, was consumed in the reaction of 2,3-BD formation. However, there still remained 0.91 and 1.1 g/L AC when glucose was depleted in the fermentation broths of BSF7 and BSF8, respectively, indicating more NADH should be provided for converting AC to 2,3-meso-BD.

It was worth noting that BSF3 produced less acetate and succinate, and showed faster glucose consumption rate than BSF5, although with 0.44 g/L lactate being produced when glucose was depleted. Considering the minimum byproducts’ yields (about 0.011 mol succinate/mol glucose, 0.054 mol acetate/mol glucose, and 0.088 mol lactate/mol glucose) of this strain, *budC* was introduced into it, yielding BSF17. For all the mutants that could produce d-(−)- or meso-2,3-BD as shown in Table 2, 2,3-BD was transformed into AC after glucose was depleted. Interestingly, the decreased amount of meso-2,3-BD produced by BSF17 (from 2.93 g/L at 37 h to 1.56 g/L at 60 h) was less than that of d-(−)-2,3-BD produced by BSF18 (*B. subtilis* 168Δ*upp*Δ*acoA*Δ*bdhA*; pHP13-*P*_*43*_-*bdhA*) (Fu et al. [25]) (3.19 g/L at 37 h–0.11 g/L at 60 h) under the same condition, which suggested that AR coded by *budC* might have a less tendency to be producing AC than AR that was coded by *bdhA* after glucose was completely consumed.

**Table 2:** Long-term fermentation of *B. subtilis* strains in the LB(Y) medium supplemented with 105 g/L glucose under microaerobic condition

Strain	CDW^a^ (g/L) (at 216 h)	2,3-BD^b^ (g/L)	AC^b^ (g/L)	Acetate^b^ (g/L)	Lactate^b^ (g/L)	Succinate^b^ (g/L)	Glucose consumed (g/h) (at 216 h)	Glucose uptake rate [g/(L h)]	2,3-BD productivity [g/(L h)]	2,3-BD yield (mol/mol)
BSF1	3.10 ± 0.38	40.84 ± 1.25(D)	3.47 ± 0.49	0.63 ± 0.11	1.19 ± 0.15	0.10 ± 0.02	87.3 ± 2.1	0.404	0.189	0.936
BSF9	3.82 ± 0.41	50.16 ± 0.52(M)	3.35 ± 0.79	0.62 ± 0.11	0.04 ± 0.00	0.16 ± 0.02	104.3 ± 0.5	0.483	0.232	0.962
BSF25	4.17 ± 0.22	49.59 ± 0.23(M)	2.66 ± 0.48	0.34 ± 0.04	1.17 ± 0.17	0.11 ± 0.02	105.0 ± 0.0	0.486	0.230	0.945

Bacteria were cultivated using a mixture of 100-mL LBR medium and 10.5 % glucose in a 250-mL flask kept agitated at a speed of 100 rpm and 37 °C; Data are average values and standard deviations of triplicate experiments. D, d-(−)-2,3-BD; M, meso-2,3-BD

^a^The CDW was calculated using the relationship: “CDW = OD_600_*0.325”

^b^These metabolites were measured at 216 h

### Improving NADH supply and distribution to convert more AC to meso-2,3-BD

In order to provide more NADH for converting AC to meso-2,3-BD, *udhA* was introduced into BSF6 by vector pPSDBUE (overexpressing *alsS*, *alsD*, *budC*, and *udhA* genes), yielding BSF9. Noticeably, BSF9 exhibited a faster glucose consumption rate than BSF4-8. With the increased NADH availability, a maximum titer of 3.56 g/L meso-2,3-BD (purity >99 %) was produced by BSF9, which was 22.8 % higher than that produced by BSF8. Meanwhile, the amount of AC of BSF9 (0.68 g/L) was 38.2 % lower than that of BSF8 (1.10 g/L). The conversion of more AC to meso-2,3-BD by BSF9 implied that more NADH was provided for the reaction of 2,3-BD formation catalyzed by AR. Although the concentration of AC decreased by degrees, there was still a quantity of AC (0.68 g/L) left in the fermentation culture of BSF9 due to the lack of NADH available, which would be solved by reducing the oxygen level in rich medium afterward. In addition, both of the byproducts, acetate and succinate, accumulated by BSF9 were decreased compared with BSF8, which might be caused by an extra copies of *alsS* and *alsD* in BSF9 compared to BSF8. As expected, BSF9 exhibited the highest meso-2,3-BD production among all the mutants. Noticeably, deleting *udhA* in *E. coli*, led to significant increases in the NADPH/NADP^+^ ratio and improved the NADPH-dependent xylose reduction [44], while in *Corynebacterium glutamicum*, the expression of the *pntAB* genes (coding a membrane-integral nicotinamide nucleotide transhydrogenase PntAB to drive the reduction of NADP^+^ via the oxidation of NADH) from *E. coli* resulted in the conversion of NADH to NADPH, which both demonstrated that the transhydrogenase had been widely used and could be a suitable strategy for cofactor engineering [45].

In addition, the pPSDBUE was also introduced into BSF3, yielding BSF25. In the absence of *pta* and *ldh* deletions, BSF25 showed an increased glucose consumption rate, and accumulated less acetate and succinate than BSF9 (although producing a small amount of lactate at 36 h). As shown in Table 1, BSF25 exhibited the fastest glucose consumption rate among all the mutants. However, BSF9 produced less AC and more meso-2,3-BD (3.56 g/L) than that of BSF25 (3.28 g/L), as the result of the further increased NADH/NAD^+^ ratio (Fig. 2) in BSF9 by deleting *ldh*. Remarkably, the AR activities in BSF17, BSF25, and BSF9 were 511.5 ± 104.4, 488.0 ± 25.3, and 367.8 ± 27.1 nmol/min^−1^ mg cell protein^−1^, respectively, in M9 medium with 10 g/L glucose in microaerobic conditions at 34 h, which were at the same level.

**Fig. 3:**
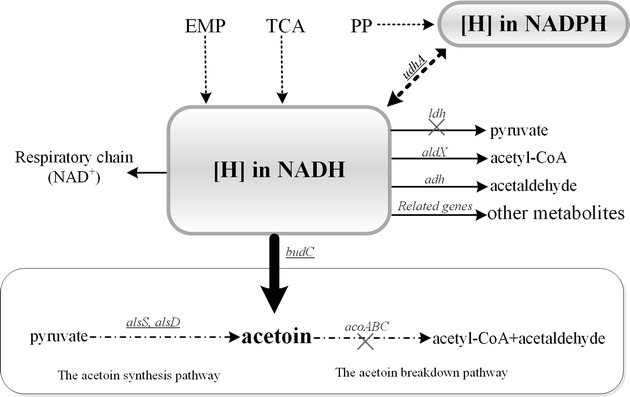
Metabolic distribution of reducing power [H] in *B. subtilis* mutants. The *arrows* represent the delivery of the [H] in NADH, and the *dashed arrows* represent the creation of the [H]. The acetoin synthesis and breakdown pathway are indicated in the *oval frame*. Overexpressed genes are *underlined*. Disrupted pathway steps ar indicated by *broken arrows*

The overall strategy used to increase the pool of NADH available and reduce its consumption (Fig. 3) was as follows. The *udhA* gene was overexpressed in BSF25 and BSF9, transforming NADPH [produced by the pentose phosphate pathway (PPP)] to NADH supply. To reduce the consumption of NADH in lactate synthetic pathway, *ldh* was deleted to block the pathway, and the deletion had increased 2,3-BD yield and titer of BSF9 (Δ*ldh*) compared with BSF25 in the M9 medium. Afterward, high volume and low agitation speed were adopted to reduce the DO level. Therefore, the wastage of [H] in NADH produced by Embden Meyerhof Parnas pathway (EMP), tricarboxylic acid cycle (TCA) and from the reoxidation of excess NADPH, could be considerably reduced in the respiratory chain. Furthermore, rich medium with more reducing substrate could allow higher NADH availability and cell density, and hence the LBR medium was available to be used subsequently, thus leading to the improved 2,3-BD production in this study.

### Fermentation in rich medium to enhance 2,3-BD production in agitating flask

The unsatisfactory 2,3-BD productivity and yield in M9 medium might be caused by two reasons. First, the biomass was very low in the stationary phase in M9 medium, resulting in a low overall glucose consumption rate and 2,3-BD productivity. Second, a high proportion of NADH was wasted in the respiratory chain, leaving considerable AC in the fermentation broth, and therefore the 2,3-BD yield appeared to be low.

In order to investigate the enhanced 2,3-BD production in the medium containing enough reducing substances, we tried to use LBR medium with 105 g/L glucose in a batch flask fermentation process (Table 2). As expected, both BSF25 (49.59 g/L, 94.5 % of the theoretical maximum yield) and BSF9 (50.16 g/L, 96.2 % of the theoretical maximum yield) showed a high meso-2,3-BD production and yield. Remarkably, the two strains produced almost the same amount of meso-2,3-BD, probably because of the sufficient availability of the reducing substances provided by the rich medium LBR. In addition, it was worth noting that the 2,3-BD production of BSF1 (without *udhA* introduced) was not satisfactory, possibly due to the low glucose consumption rate or the imbalance of the reducing capacity under microaerobic condition (Table 2).

### High 2,3-BD production achieved by optimization of oxygen supply in 5-L fermentor and its advantages using *B. subtilis* as the producer

**Fig. 4:**
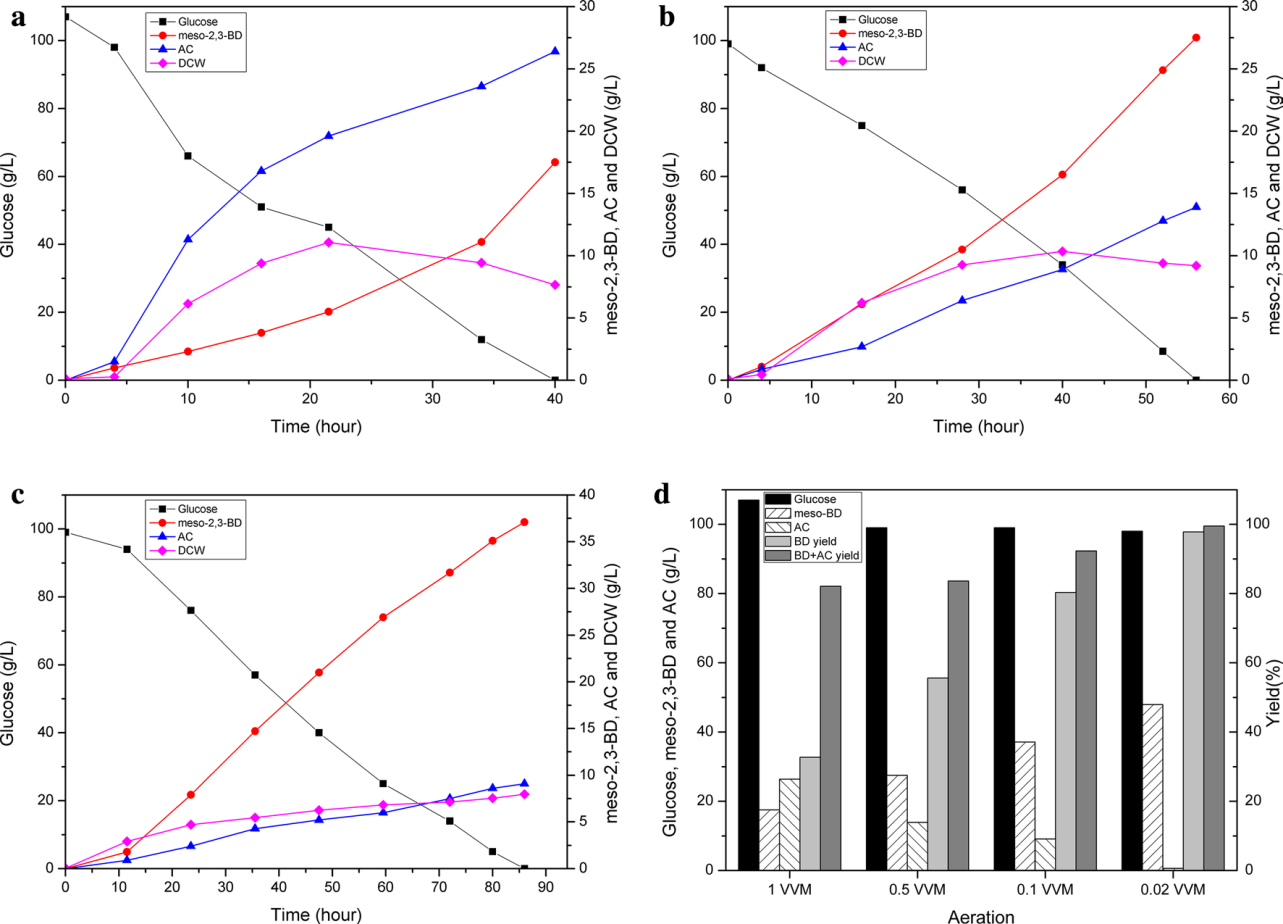
Optimization of oxygen supply in 5-L fermentor. BSF9 was cultured in LBR medium with glucose at 37 °C and 300 rpm in a 5-L fermenter at different aeration rates. *Filled square* glucose concentration; *Filled circle* 2,3-BD concentration; *Filled triangle* AC concentration; *Filled diamond* cell density, optical density at 600 nm (OD600). Cultivation was carried out at an initial pH of 6.5. Agitation speed was 300 rpm. Batch fermentation with aeration rates of **a** 1 vvm; **b** 0.5 vvm; **c** 0.1 vvm; **d** glucose consumption, meso-2,3-BD production, AC, meso-2,3-BD yield, and AC + BD total yield of BSF9 during oxygen-supply optimization in batch fermentation process

To improve the meso-2,3-BD titer and productivity and achieve a more appropriate

fermentation condition, batch fermentation was conducted with different DO levels. As shown in Fig. 4a, b, and c, the aeration rate strongly influenced extracellular metabolite concentrations during 2,3-BD fermentation. It was indicated that the meso-2,3-BD yield increased from 0.164 to 0.489 g/g glucose as the aeration rate decreased from 1–0.02 VVM (Fig. 4d). *Bacillus subtilis* was considered to be an aerobic bacterium, and under aerobic conditions, it could secrete industrial enzymes [24], riboflavin [46, 47], AC [33, 48], etc. However, as for chiral meso-2,3-BD production using *B. subtilis*, limited oxygen condition was preferred for the transaction from AC to BD. As a result, the aeration rate as low as 0.02 VVM was used subsequently to achieve high meso-2,3-BD yield and production.

**Fig. 5:**
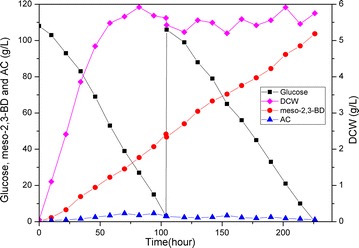
Meso-2,3-BD production from glucose using BSF9 in fed-batch fermentation. *Filled square* glucose concentration; *Filled circle* 2,3-BD concentration; *Filled triangle* AC concentration; *Filled diamond* cell density, optical density at 600 nm (OD600). Cultivation was carried out at an initial pH of 6.5; during the fermentation processes, the pH was uncontrolled. Agitation speed was 300 rpm, and aeration rate was 0.02 vvm

Under the optimized fermentation conditions, a final titer of 103.7 g/L meso-2,3-BD (purity >99 %) and 1.1 g/L AC as the main byproducts were achieved by BSF9 (Fig. 5). The meso-2,3-BD productivity was 0.459 g/(L h), which was almost twice as much as that in flask experiments. No more than 0.2 g/L succinate or acetate was produced. No pyruvic, α-ketoglutaric acid, diacetyl, and lactate were detected. During the fermentation, the biomass increased slowly in the first 50 h and was maintained at the level of approximately 5.2 g/L CDW until the end. This low oxygen level resulted in a low biomass and a slow glucose consumption rate, which might account for the unsatisfactory meso-2,3-BD productivity. However, no more than 1.1 g/L AC was produced under this condition, which not only caused a high yield of 0.487 g meso-2,3-BD/g glucose (97.4 % of the theoretical maximum yield), but also would be beneficial for the downstream purification process.

Significantly, even in the absence of the pH control, BSF9 could maintain a good stability of 2,3-BD production consistently for over 200 h. The pH fell to 5.8 during the first 10 h, gradually increased to 6.4 in the next 24 h, and then maintained at 6.4 until the end of the fermentation, which indicates the robustness of the mutant. Overall, the low aeration rate (0.02 VVM) and agitation speed (300 rpm), as well as simple fed-batch process, could reduce the costs of energy and operation process for industrial-level production.

### The ability of *B. subtilis* for efficient 2,3-BD production at higher temperature

**Fig. 6:**
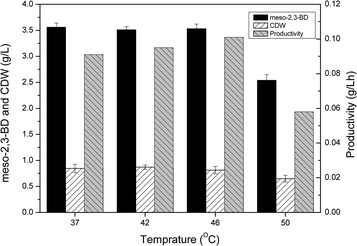
Meso-2,3-BD production, biomass, and productivity using BSF9 at different fermentation temperatures. BSF9 was cultivated in a mixture of 100 mL M9 and 10 g/L glucose in a 250-mL flask kept agitated at a speed of 100 rpm

Compared to most of 2,3-BD producers which mainly functioned below 37 °C (e.g., *S. cerevisiae* at 30 °C [20, 21]), *B. subtilis* could grow well and had the potential to produce 2,3-BD efficiently at a higher temperature. Considering this possibility, we tested the 2,3-BD production ability of BSF9 at 37, 42, 46, and 50 °C, respectively. As shown in Fig. 6, in the minimal medium, the titer of meso-2,3-BD was almost unchanged at 37, 42, and 46 °C, while the meso-2,3-BD productivity increased when the cultivation temperature was increased from 37 to 46 °C. However, the titer and productivity at 50 °C decreased by 28.6 and 36.3 % compared to those at 37 °C, respectively, which can be attributed to the much lower biomass and about 0.8 g/L acetate produced at this temperature. This problem could be got rid of with the addition of a small amount of organic nitrogen sources (e.g., 5 g/L corn steep liquor powder or yeast power) to obtain a satisfactory meso-2,3-BD production and yield. (data not shown).

Overall, strain BSF9 exhibited the ability of producing 2,3-BD efficiently at a higher temperature above 46 °C, which allowed reduction in cooling costs during fermentation process, especially when the ambient temperature was relatively high. However, in the case of 2,3-BD production at 50 °C, the growth of this strain still needs to be improved by adaptive evolution in the future study.

### Phylogenetic tree based on typical amino acid sequences of AR in 2,3-BD producers

**Fig. 7:**
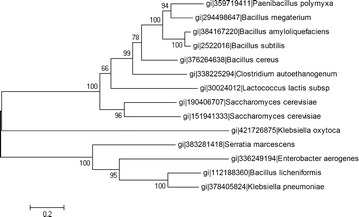
The phylogenetic tree based on typical amino acid sequences of AR from 14 2,3-BD producers. The sequences were extracted from the NCBI protein database by a BLAST search. The phylogenetic tree was constructed by the neighbor-joining method with the Poisson correction model by the software MEGA 5

The formation mechanisms of 2,3-BD isomers had been the subject of much debate [2], and a preferred model for the formation of 2,3-BD isomers was proposed in consideration of the existence of different BDHs/ARs [49, 50]. To investigate the relationship between different ARs and microorganisms, the phylogenetic tree was built based on the typical amino acid sequences of AR of part of the 2,3-BD producers (Fig. 7). The typical AR amino acid sequences expressed in *B. amyloliquefaciens*, *P. polymyxa*, and *B. subtilis* exhibited remarkable similarity, which was consistent with their close relationship in biological evolution. This phenomenon also occurred in the cases of *K. pneumonia* and *K. oxytoca*. These results suggested that the wild-type microorganisms with close relationship in biological evolution might possess (at least) one kind of AR with high identity of typical amino acid sequence.

*B. subtilis* had been reported to produce a mixture of meso- and d-(−)-2,3-BD for a long time [5]. In our previous study, wild-type *B. subtilis* 168 was found generating only d-(−)-2,3-BD (purity >99 %) under low oxygen conditions, while in the present study, *B. subtilis* 168 was first successfully engineered to produce chiral meso-2,3-BD (purity >99 %). Noticeably, d-(−)-2,3-BD dehydrogenase coded by *bdhA* was thought to be the major AR in *B. subtilis,* and its deletion abolished 2,3-BD production in early stationary phase [28, 51]; however, the other 2,3-BD dehydrogenases remained unknown. In a recent study [52], meso-2,3-BD dehydrogenase was identified from a strain of *B. subtilis*, and it was suggested that a nonconservative amino acid substitution from Asp to Gly of this dehydrogenase caused the loss of its catalytic function and enzymatic activity. This result might further illustrate the fact that wild-type *B. subtilis* 168 generated only d-(−)-2,3-BD (purity >99 %). Interestingly, *B. licheniformis*, which had a close relationship with *B. subtilis*, was found to produce d-(−)- and meso-2,3-BD with a ratio of nearly 1:1 [53], and a genetically modified strain for d-(−)-2,3-BD production was obtained by deleting the meso-2,3-BD dehydrogenase coded by *budC* [54]. It should be noticed that the typical amino acid sequence of AR in *B. licheniformis* [d-(−)- and meso-2,3-BD as major products] and that of AR *K. pneumonia* [meso-2,3-BD and L-(+)-2,3-BD with a ratio of 9:1] had high similarity, although those two strains produced different kinds of 2,3-BD and had a far evolutionary distance between them. Moreover, it had been reported that the glycerol dehydrogenase coded by *gdh* in *B. licheniformis* could catalyze interconversion between (3R)-AC and d-(−)-2,3-BD [53], although the protein sequence of glycerol dehydrogenase was dissimilar from that of AR coded by bdhA in *B. subtilis*. These results inspired the researchers to reinvestigate the chiral configuration of 2,3-BD mixtures and the enzymes that catalyze the reaction between AC and 2,3-BD isomers.

## Conclusions

In summary, the *bdhA* in *B. subtilis* 168 was replaced by *budC* from *K. pneumonia* CICC10011 and resulted in chiral meso-2,3-BD production. The production of chiral meso-isomer obtained in this work was the highest among the reported *Bacillus* genuses, which were excellent candidates for industrial-scale microbial fermentation of meso-2,3-BD. This study provided a deep understanding of chiral 2,3-BD metabolism in *B. subtilis*, and a strategy for enhancing the chiral pure 2,3-BD production through genetic modification. Moreover, deletion of *ldh* and introduction of *udhA*, low DO control, and additional reducing substrates further enhanced meso-2,3-BD production, which indicated that the key factor for meso-2,3-BD production in *B. subtilis* was transformation of AC to 2,3-BD. Finally, 103.7 g/L meso-2,3-BD with a yield of 0.974 mol/mol glucose was obtained by the optimal strain BSF9 under limited oxygen conditions. The simple operation involving, e.g., uncontrolled pH, low aeration rate, and low agitation speed, could reduce the costs of energy and operational process for industrial-level production. The fermentation process exhibited high yield, titer, and purity of meso-2,3-BD, demonstrating that *B. subtilis* as a Class I microorganism was a competitive candidate as a meso-2,3-BD producer and could be further developed for its production at industrial level.

## Methods

### Bacteria strains, media, and growth conditions

All *B. subtilis* strains used were derived from the wild-type *B. subtilis* 168. The strains and plasmids used in this work are listed in Table 3.

**Table 3:** Strains and plasmids

Name	Relevant genotype	Source/reference
Strains		
* B. subtilis* 168	Wide-type strain, trpC2	BGSC^a^
* E. coli* DH5α		Invitrogen
*K. pneumoniae* CICC 10011		CICC^b^
BSF1	*B. subtilis* 168Δ*upp*	(Fu et al. [25])
BSF2	*B. subtilis* 168Δ*upp*Δ*acoA*	This study
BSF3	*B. subtilis* 168Δ*upp*Δ*acoA*Δ*bdhA*	(Fu et al. [25])
BSF4	*B. subtilis* 168Δ*upp*Δ*acoA*Δ*bdhA*Δ*pta*	This study
BSF5	*B. subtilis* 168Δ*upp*Δ*acoA*Δ*bdhA*Δ*pta*Δ*ldh*	This study
BSF6	*B. subtilis* 168Δ*upp*Δ*acoA*Δ*bdhA*Δ*pta*Δ*ldh*; *P*_*43*_::*alsS*-*alsD*	This study
BSF7	*B. subtilis* 168Δ*upp*Δ*acoA*Δ*bdhA*Δ*pta*Δ*ldh*; *P*_*43*_::*budC*, *spc*	This study
BSF8	*B. subtilis* 168Δ*upp*Δ*acoA*Δ*bdhA*Δ*pta*Δ*ldh*; *P*_*43*_::*alsS*-*alsD*; *P*_*43*_::*budC*, *spc*	This study
BSF9	*B. subtilis* 168Δ*upp*Δ*acoA*Δ*bdhA*Δ*pta*Δ*ldh*; *P*_*43*_::*alsS*-*alsD*; *P*_*43*_::*alsS*-*alsD*-*budC*-*udhA*, *erm*	This study
BSF17	*B. subtilis* 168Δ*upp*Δ*acoA*Δ*bdhA*; pHP13-*P*_*43*_-*budC*	This study
BSF18	*B. subtilis* 168Δ*upp*Δ*acoA*Δ*bdhA*; pHP13-*P*_*43*_-*bdhA*	(Fu et al. [25])
BSF25	*B. subtilis* 168Δ*upp*Δ*acoA*Δ*bdhA*; *P*_*43*_::*alsS*-*alsD*-*budC*-*udhA*, *erm*	This study
Plasmids		
pUC18	Amp^r^	Lab Stock
pUC18-*upp*FB-*neo*	Kan^r^	Lab Stock
pC194	Cm^r^, *Bacillus* cloning vector	Lab Stock
pE194	Em^r^, *Bacillus* cloning vector	Lab Stock
pHP13	Cm^r^, Em^r^, *E. coli*–*B. subtilis* shuttle vector	Lab Stock
pUC18-SP	Amp^r^, Spc^r^, containing *P*_*43*_ promoter	Lab stock
pUC18-SP-*budC*	Amp^r^, Spc^r^, containing *P*_*43*_ promoter and *budC*	This study
pCU	puC18 containing *cat*-*upp* cassette	Lab stock
pCU-*pta*FB	pCU containing *pta* flanks	This study
pCU-*ldh*FB	pCU containing *ldh* flanks	This study
pCU-*bdhA*FB-PSD	pCU-*bdhA*FB containing *alsS*-*alsD* under *P*_*43*_	This study
pUC18-SP-*budC*	Amp^r^, Spc^r^, containing *budC* under *P*_*43*_ promoter	This study
pUC18-*P*_*43*_	Amp^r^, Em^r^, containing *P*_*43*_ promoter	This study
pUC18-*P*_*43*_-SD	Amp^r^, Em^r^, containing *alsS* and *alsD* under *P*_*43*_ promoter	This study
pUC18-*P*_*43*_-SDU	Amp^r^, Em^r^, containing *alsS*, *alsD* and *udhA* under *P*_*43*_ promoter	This study
pUC18-*P*_*43*_-SDBU	Amp^r^, Em^r^, containing *alsS*, *alsD*, *budC*, and *udhA* under *P*_*43*_ promoter	This study
pPSDBUE	pUC18-*P*_*43*_-SDBU containing erythromycin resistance gene	This study
pHP13-*P*_*43*_-*budC*	Em^r^, Cm^r^, containing *budC* under *P*_*43*_ promoter	This study

^a^ *Bacillus* Genetic Stock Center

^b^China Center of Industrial Culture Collection

The Luria–Bertani (LB) medium was used for plasmid construction in *E. coli*. The M9 medium plus glucose was used as the minimum medium for flask fermentations to test the physiological characterizations of the engineered strains. Tryptophan (50 mg/L) was added in M9 medium for all *B. subtilis* strains. The 5FU medium [39] was used for the selection of marker-free engineered colonies of *B. subtilis*. The LBR medium plus glucose as a rich medium was used for the high concentration of sugar fermentation, which contained 3 g/L tryptone, 15 g/L yeast extract, 3 g/L NaCl, and appropriate glucose. Antibiotics were added appropriately (for *B. subtilis*, chloramphenicol 5 mg/mL, erythromycin 3 mg/mL, spectinomycin 100 mg/mL, neomycin 5 mg/mL; for *E. coli*, ampicillin 100 mg/mL; chloramphenicol 15 mg/mL, and spectinomycin 100 mg/mL). With the exception of *B. subtilis* 168, 5 mg/mL neomycin was added to the medium for all the *B. subtilis* mutants.

Strains were stored at −80 °C and revived by growing on LB agar slants/plates. Single colonies were transferred into 5 mL LB medium containing the corresponding antibiotic when required. All cultivations were performed at 37 °C. After 12-h incubation at 220 rpm, a 2 % (v/v) inocula was added to 50 mL M9 medium with 10 g/L glucose or LBR medium with 105 g/L glucose, in a 250-mL shake flask and cultivated for 12 h at 220 rpm to prepare the M9 or LBR seed, respectively. Cell growth was determined by measuring turbidity at 600 nm (OD_600_) using a UV–Vis spectrophotometer. This M9 or LBR seed culture was inoculated appropriately to set an initial OD_600_ of 0.05 under cultured conditions for flask fermentation, and 100 mL corresponding culture in a 250-mL flask with silica gel plug kept agitated at a speed of 100 rpm was used for the microaerobic tests. For batch fermentation, the LBR medium aforementioned was inoculated with 100 mL LBR seed culture in a 5-L fermenter (Bailun, Shanghai, China) with an operating volume of about 2.3 L. All cultivations were carried out at 37 °C with different aeration rates. The agitation speed was maintained at 300 rpm. The initial pH of the medium was 6.5, and during the fermentation process, the pH value was uncontrolled. The initial glucose concentration was ~110 g/L. For fed-batch fermentation, the inoculation procedure and fermentation conditions were identical to that described for batch experiments, and 250-mL 1000 g/L glucose stock and 5 g yeast extract power were added when the residual glucose concentration was reduced to about 10 g/L.

### Plasmid construction

All primers are listed in Table 4.

**Table 4:** Primers used in this study

Primer name	Sequence (5′–3′)
d-*pta*-FU	ACATGCATGCAATTGAAGGACATCATCGCTA
d-*pta*-FL	CATTGTCTTCAATTCTCGAGCGGCCATGGGGACTTAAGAACCTCCTCAAAAAGTTACAA
d-*pta*-BU	TTTTGAGGAGGTTCTTAAGTCCCCATGGCCGCTCGAGAATTGAAGACAATGGCAGCTCT
d-*pta*-BL	CGGGGTACCACAGCTTGCGCTTTAACGAGA
d-*pta*-FsnU	ACATGCATGCCCCACTCGTAATCGTCAAAGCC
d-*pta*-FsnL	CGGGGTACCAAGTCCCATGCCGTTAATCACC
d-*ldh*-FU	TGGACAGCCTGAGGAACTCTCG
d-*ldh*-FL	ACGTCATCTGCACTCGAGAGATCTCTTAAGTCCATCACATCGCCCATTGCTT
d-*ldh*-BU	CGATGTGATGGACTTAAGAGATCTCTCGAGTGCAGATGACGTGTACATCGG
d-*ldh*-BL	CAGGAAGGCTCCAGATGGTGA
d-*ldh*-FsnU	GCGCGGGACGTCGGCAAATGCAGGTTCATCCTC
d-*ldh*-FsnL	GCGCGGGACGTCCCTCGCGGTAAATCTCTTTCGG
S-*budC*-U	CGCGGATCCCCAAGGAGGGTATAGCTATGAAAAAAGTCGCACTTGTTACC
S-*budC*-L	TGCTCTAGAGGATGCTTACACTTACACTCAAAC
O-*P*_*43*_-U	CCGGAATTCCAGGCCGGGGCATATGGGAAACA
O-*P*_*43*_-L	CGCGGATCCTCCCCCGGGAATTCTTGTCTGTTCATGTGTA
O-*alsSD*-U	TCCCCCGGGCCAAGGAGGGTATAGCTTTGACAAAAGCAACAAAAGAACAA
O-*alsSD*-L	CGCGGATCCTTATTCAGGGCTTCCTTCAGTTGT
O-*budC*-U	CGCGGATCCCCAAGGAGGGTATAGCTATGAAAAAAGTCGCACTTGTTACCG
O-*budC*-L	CGCGGATCCGCCTAGCTAGCCGGACTAGTTTAGTTAAATACCATCCCGCCGTC
O-*bdhA*-U	CGCGGATCCAAAGGAGGAATTCAAAATGAAGGCAGCAAGATGGCATAAC
O-*bdhA*-L	CGCGGATCCGCACTCGAGCGGACTTAAGCAAATTAGTTAGGTCTAACAAG
O-*udhA*-U	TTAGACTAGTACAAGGAGGACCCTACCATGCCACATTC
O-*udhA*-L	ACATGCATGCGCCTAGCTAGCCGGCGACGCGTTTAAAACAGGCGGTTTAAACCG
O-*em*-U	GCGCGGGACGTCCCCACTTTATCCAATTTTCGTT
O-*em*-L	GCGCGGGACGTCCTCACGTTAAGGGATTTTGGTCA
E-*budC*-U	TCGGGGTACCAAAGGAGGAATTCAAAATGAAAAAAGTCGCACTTGTTACCG
E-*budC*-L	CGCGGATCCGCACTCGAGCGGACTTAAGTTAGTTAAATACCATCCCGCCGTC

For *pta* (accession number, BG10634) deletion, the upstream and downstream fragments (*pta*-F and *pta*-B—forward and backward fragments) were amplified from the *B. subtilis* genome using primers, d-*pta*-FU/L and d-*pta*-BU/L, respectively. The two fragments were fused and amplified by fusion PCR with primers d-*pta*-FsnU/L. The fused fragment (*pta*-FB) was then digested with *Sph*I-*Kpn*I, and was ligated into the same digested sites of pCU to create pCU-*pta*FB. Using a similar procedure, the *ldh*FB-fused fragments were amplified with corresponding primers for *ldh* (accession number, BG19003) deletion. Digested with *Aat*II, the fragment was ligated into the corresponding site of pCU to yield plasmid pCU-*ldh*FB. For marker-free gene overexpression, the *B. subtilis* strong promoter *P*_*43*_ and the fragments of *alsS* and *alsD* genes were amplified together by primers O-*P*_*43*_-U and O-*alsSD*-L from pPSDBUE, and then placed between the upstream and downstream fragments of *bdhA* in pCU-*bdhA*FB, generating pCU-*bdhA*-PSD for the overexpression of *alsSD* in the genome of *B. subtilis*.

The vector pPSDBUE, for overexpressions of *alsS* (accession number, BG10471), *alsD* (accession number, BG10472), *budC* (accession number, HM113375), and *udhA* (accession number, EG11428) in the genome of *B. subtilis* by single-crossing event at *P*_*43*_ plot, was built as follows. The *P*_*43*_ promoter was amplified from the *B. subtilis* 168 chromosomal DNA by the primers O-*P*_*43*_-U/L, digested with *Bam*HI-*Sma*I, and ligated to the matching sites of pUC18, yielding pUC18-*P*_*43*_. Then the *alsS* and *alsD* genes were amplified together by the primers O-*alsSD*-U/L, digested with *Sma*I-*Bam*HI, and ligated to pUC18-*P*_*43*_ that digested with the same enzyme to create pUC18-*P*_*43*_-SD. Next, the *udhA* gene was amplified using primers O-*udhA*-U/L, digested with *Xba*I-*Sal*I and ligated to the same sites of pUC18-*P*_*43*_-SD to create pUC18-*P*_*43*_-SDU. Subsequently, the *budC* gene amplified by the primers O-*budC*-U/L was digested with *Bam*HI and ligated to the pUC18-*P*_*43*_-SDU digested with the same restriction site, yielding pUC18-*P*_*43*_-SDBU. Finally, the erythromycin resistance gene *erm* were amplified from the pHP13 by the primers O-em-U/L, and ligated to the *Aat*II-digested pUC18-*P*_*43*_-SDBU, yielding pPSDBUE.

The shuttle vectors pHP13-*P*_*43*_-*budC* was constructed for *budC* overexpression, based on the *B. subtilis*-*E. coli* shuttle vector pHP13-*P*_*43*_ with the *B. subtilis* strong promoter *P*_*43*_. The *budC* was amplified by PCR from *K. pneumoniae* CICC10011 with primers E-*budC*-U/L. These fragments were digested with the restriction sites *Kpn*I-*Bam*HI, and ligated to *Kpn*I-*Bam*HI digested pHP13-*P*_*43*_, yielding pHP13-*P*_*43*_-*budC*.

The pUC18-SP vector for *budC* overexpression in the genome of *B. subtilis* can be selected by the spectinomycin. The *budC* gene was amplified by primers S-*budC*-U/L, digested with *Bam*HI-*Xba*I, and ligated to the matching sites of pUC18-SP to create pUC18-SP-*budC*.

### Strains’ construction

The vector pCU-*acoA*FB (*acoA* accession number, BG12558) was integrated into BSF1 chromosome by the first single-crossover recombination, and chloramphenicol-resistant transformants were selected and verified by PCR. Next, the resulting transformant was cultured in LB liquid medium for 12 h, and then the cells were spread on a 5FU agar plate. The *acoA*-deleted strain denoted as BSF2 was selected from the grown colonies by PCR verification using primers d-*acoA*-FU/BL. The *pta* and *ldh* genes were knocked out step by step using the same method (Additional file 1: Figure S1), yielding BSF4 and BSF5. Subsequently, the plasmid pCU-*bdhA*FB-PSD (*bdhA* accession number, BG12803) was introduced into the BSF5 strain for *alsSD* operon overexpression using the two-step method described above, yielding BSF6 (Additional file 2: Figure S2).

The shuttle vector for overexpressing the *budC* was transformed into the *B. subtilis* strains, yielding BSF17. The vector pUC18-SP-*budC* was integrated into the choromosomes of BSF5 and BSF6, yielding BSF7 and BSF8, respectively. The vector pPSDBUE was integrated into the choromosomes of BSF3 and BSF6 at *P*_*43*_ plot, yielding BSF25 and BSF9, respectively.

### AR enzyme assay

To determine the levels of AR activities, bacteria were cultured in the M9 medium under the microaerobic condition for 20 h during exponential growth. AR activity was determined by measuring pyruvate linked NADH oxidation at 340 nm as described previously [51, 55], except for the reaction temperature set at 37 °C. Cells were harvested from 1 mL culture samples by centrifugation for 1 min, and the supernatant was removed. The precipitate was then broken in 400 μL 0.1 M phosphate buffer (pH = 7.0) by sonication (130 W, 20 kHz, pulse: 5 s on; 5 s off; total: 20 min) followed by centrifugation at 4 °C to remove cell debris. AR activity was assayed spectrophotometrically at 37 °C in a total volume of 1 mL 0.1 M phosphate buffer (pH = 7) containing 0.2 mM NADH and 50 mM AC. The reaction was initiated by the addition of 20 μL cell extract and monitored by the decrease in absorbance at 340 nm. Total protein concentrations were determined according to the Bradford method [56].

### Detection of metabolites

Cell growth was monitored by measuring optical density at 600 nm (OD_600_). Glucose was determined using a glucose analyzer. Extracellular metabolite concentrations were determined by HPLC [33]. The mobile phase consisted of 5 mM H_2_SO_4_ with the flow rate at 0.4 ml/min, and the column temperature was set at 65 °C. To determine the enantiomeric distribution of 2,3-BD, the fermentation samples were saturated with sodium chloride and extracted with ethyl acetate. The isomers in the extracts were then analyzed by GC-FID (Hewlett Packard) equipped with a HP-chiral 20b column as described previously [57]. The oven temperature program was as follows: 40 °C (2 min), increased to 75 °C (4 min) at 5 °C min^−1^, followed by a ramp of 1 °C min^−1^ to 80 °C (2 min), and finally, 15 °C min^−1^ to 230 °C (4 min). The intracellular NADH and NAD^+^ levels were determined by NAD/NADH Quantitation Kit (SIGMA).

## Additional files

10.1186/s13068-016-0502-5 A simple method for marker-free genes knockout.
10.1186/s13068-016-0502-5 The principle for the *alsS* and *alsD* marker-free overexpression.

## Abbreviations

AC: acetoin
AR: acetoin reductase
BD: butanediol
BDH: butanediol dehydrogenase
DO: dissolved oxygen
EMP: Embden Meyerhof Parnas pathway
GRAS: generally regarded as safe
LB: Luria–Bertani
OD: optical density
ppb: parts per billion
PPP: pentose phosphate pathway
TCA: tricarboxylic acid cycle
