# Supplementary figures and images for: Metabolic engineering of Bacillus subtilis for chiral pure meso-2,3-butanediol production
Source: Biotechnol Biofuels. 2016 Apr 19;9:90. doi: 10.1186/s13068-016-0502-5 (PMC4837526; doi:10.1186/s13068-016-0502-5)

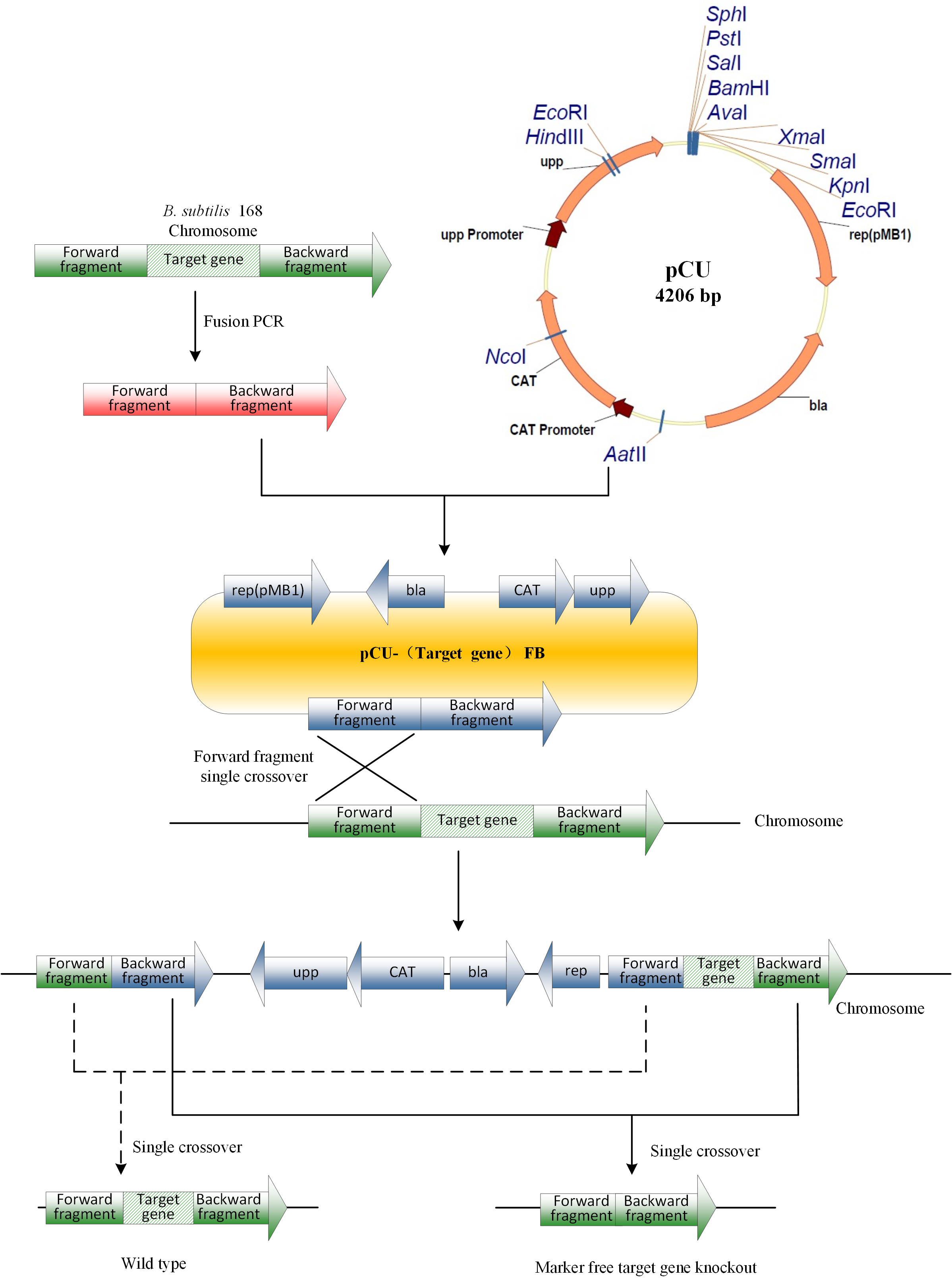

Supplement: Supplementary file 1 — 10.1186/s13068-016-0502-5 A simple method for marker-free genes knockout. [file 13068_2016_502_MOESM1_ESM.tif]

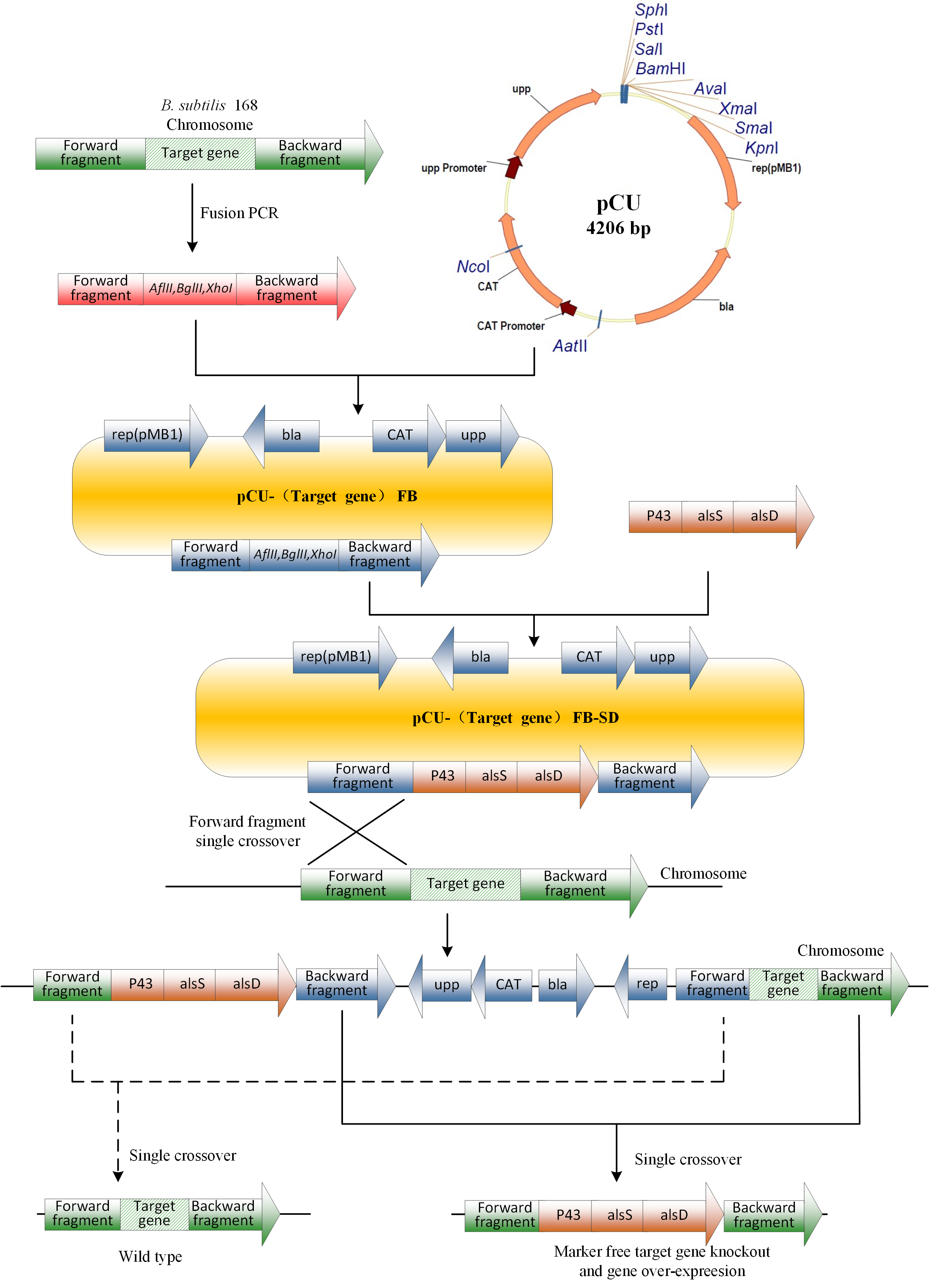

Supplement: Supplementary file 2 — 10.1186/s13068-016-0502-5 The principle for the alsS and alsD marker-free overexpression. [file 13068_2016_502_MOESM2_ESM.tif]
